# The health and wellbeing needs of veterans: a rapid review

**DOI:** 10.1186/s12888-017-1547-0

**Published:** 2017-12-29

**Authors:** Candice Oster, Andrea Morello, Anthony Venning, Paula Redpath, Sharon Lawn

**Affiliations:** Flinders Human Behaviour and Health Research Unit, Department of Psychiatry, Flinders University, GPO Box 2100, Adelaide, South Australia 5001 Australia

**Keywords:** Veteran, Rapid review, Health, Mental health, Social care

## Abstract

**Background:**

For the majority of serving members, life in the military has a positive effect on wellbeing. However, the type, intensity and duration of service, along with the transition from fulltime military to civilian life, may have a negative effect on veterans’ wellbeing. Such negative consequences, alongside the growing veteran population, indicate the need for greater exploration of veterans’ physical, mental and social wellbeing.

**Methods:**

The current paper reports on the findings of a rapid review of the literature on the health and wellbeing needs of veterans, commissioned by the Australian Department of Veterans’ Affairs to inform future programs and services. The databases Embase, Medline, Cinahl, PubMed, Web of Science and Cochrane Database were searched for systematic reviews reporting on veterans’ physical, mental and social wellbeing published in English in peer-reviewed journals.

**Results:**

A total of 21 systematic reviews were included. The reviews reported on a range of mental, physical and social health problems affecting veterans. While there was limited information on prevalence rates of physical, mental and social health problems in veterans compared to civilian populations, the reviews demonstrated the interconnection between these domains and the effect of demographic and military service factors.

**Conclusions:**

A key finding of the review is the interconnection of the mental, physical, and social health of veterans, highlighting the importance that an integrated approach to veterans’ wellbeing is adopted. It is suggested that understanding key factors, such as demographic factors and factors relating to military service, can support improved service provision for veterans.

**Electronic supplementary material:**

The online version of this article (10.1186/s12888-017-1547-0) contains supplementary material, which is available to authorized users.

## Background

Veterans (defined here as ex-members of the armed forces) are a growing population in Australia, the United Kingdom (UK) and other countries, particularly since the return of veterans from the Iraq and Afghanistan conflicts [1–3]. In the United States (US), for example, the Department of Defence estimates that there will be over 200,000 new veterans each year [4]. Serving in the armed forces potentially affects many aspects of a person’s life. For the majority of serving members, life in the armed forces has a positive effect on wellbeing [5]. However, some members will leave the armed forces facing health and wellbeing needs related to their military service [6–8].

Veterans have been observed to have a lower mortality risk relative to the general population. This has been referred to as the ‘healthy soldier effect’, resulting from the high physical health standards of entry into the armed forces [5]. However, more recent research suggests an erosion of the healthy soldier effect in veterans of contemporary conflicts [9]. Bollinger et al. [9] found US veterans of Iraq and Afghanistan had equivalent or higher expected mortality when compared to the general US population. The authors were unable to determine the reasons for this change in mortality, but postulated that it may result from prolonged and repeated deployments, survival from injuries that would have resulted in death in previous conflicts and/or a strong reliance on Guard and Reserve forces.

The healthcare needs of veterans are complex [10, 11], related to both their experiences as serving members and also the unique psychosocial issues associated with transitioning to civilian life [12, 13]. A number of factors associated with military service may contribute to the development of mental, physical and social health problems in veterans. These include the intensive physical activity associated with military life, lifestyle factors such as cigarette smoking and alcohol consumption, physical trauma, psychological trauma, viruses and exposure to toxic substances [14]. The transition to civilian life itself has also been identified as potentially problematic [12]. In the US, the Washington Post/Kaiser Family Foundation study [13] found that in a sample of 800 veterans, 70% felt that the general community misunderstood their experience. Many felt disconnected from civilian life due to profound differences between civilian and military life.

Within the structured environment of the Australian Defence Force, as in most countries, fulltime members enjoy unrestricted access to health care services that cater to the general wellbeing needs of individuals. Following transition from the defence force, veterans may need to adjust to a new, and less structured, environment, which may not provide them with the same support provided while serving. In 2015 the Australian Department of Veterans’ Affairs (DVA) commissioned a rapid review of the literature on the health and wellbeing needs of veterans in order to inform future programs and services. The World Health Organization [15] defines health across three attributes, namely physical, mental and social wellbeing. While a number of studies and reviews of the literature exploring veterans’ health have been conducted, no reviews were identified that focus on veterans’ wellbeing across all three attributes. Given the complexity of the issues facing the growing number of veterans, we sought to capture the scope of the issues across the attributes of mental, physical and social health by undertaking a rapid review of the literature.

## Method

The decision to undertake a rapid review of the literature was based on the needs of the knowledge user (in this case, the DVA) for rapid access to current knowledge on the topic. While rapid reviews are undertaken more quickly than systematic reviews, and limit aspects of the review process (e.g., use of grey literature), they are reported to produce similar conclusions to systematic reviews of the literature [16]. We followed the rapid review process outlined by Khangura et al. [16] in order to develop an ‘evidence summary’ - an overview of the available evidence about the health and wellbeing needs of veterans. The stages were as follows: (1) Needs assessment; (2) Question development and refinement; (3) Proposal development and approval; (4) Systematic literature search; (5) Screening and selection of studies; (6) Narrative synthesis of included studies; (7) Report production; (8) Ongoing follow-up and dialogue with knowledge users.

The needs assessment began with a proposal by the DVA for an evidence synthesis on the health and wellbeing needs of veterans. Consultations with the DVA were undertaken to further refine the scope of the project and gather information on their needs and interests. The needs assessment then informed the next stage of the process, where the questions were developed and refined. The final questions for the review were:

1. What are the mental, physical and social wellbeing needs of veterans?
2. What are the factors associated with the mental, physical and social wellbeing needs of veterans?

With these questions in mind, a literature search was conducted using an a priori review protocol. Following Khangura et al. [16], the decision was made to draw the information exclusively from evidence reported in systematic reviews. Systematic reviews are considered to be the highest form of evidence available [16], and focussing on systematic reviews allowed the review to be undertaken in a timely manner, reflecting the nature and purpose of a rapid review and the needs of the DVA. The search was further limited to peer-reviewed journal articles published in English.

The following databases and search terms were used:

- Embase & Medline: ‘veteran’/mj AND ‘systematic review’/de
- CINAHL: ‘veteran’ AND ‘systematic review’; delimit to ‘Academic Journals’
- PubMed: veterans health AND (Review[ptyp] AND Humans[Mesh]) AND systematic AND (Review[ptyp] AND Humans[Mesh])
- Web of Science: veteran AND ‘systematic review’
- Cochrane Systematic Reviews: veteran

The term ‘veteran’ was used as the primary population search term because it is the Medical Subject Headings (MeSH) term relating to former members of the armed services. No synonyms (such as ‘ex-forces’) were used because in a test search these were found to yield high numbers of unrelated results, which is prohibitive in a rapid review. Given that the review was focussed on all aspects of veterans’ wellbeing, the decision was made to initially search for all systematic reviews relating to veterans. Had this yielded a prohibitively large number of results then the search would have been further delimited using date limits and, if necessary, adding search terms relating to physical, mental and social wellbeing. The initial search was conducted in July 2015 and updated in September–October 2016.

Inclusion criteria for the review were: systematic review of the literature; includes military veterans who are no longer in active service; explores the physical, mental and/or social health of veterans; published in English; peer reviewed. Exclusion criteria were: non-systematic review; systematic review protocol; does not include veterans; focussed on a health care intervention; published in a language other than English.

## Results

The search yielded 386 abstracts, reduced to 320 after removal of duplicates. An abstract screening form was developed for screening against the inclusion and exclusion criteria [17]. The form was piloted with 20 abstracts reviewed by two authors (CO and AV) to ensure that both authors understood the criteria. Each author then reviewed half of the remaining abstracts (150 each) against the screening form. Articles where the reviewers were unsure if they should be included were discussed and consensus reached.

A total of 39 articles were identified for full-text collection and assessed again for eligibility against the selection criteria. Eleven articles were excluded at this stage because they were: a systematic review protocol (*n* = 2), not a systematic review (*n* = 1), could not access full text within the timeframe (n = 1), did not include veterans in the study population (n = 2), were focussed on interventions (*n* = 4) or were focussed on diagnosis and treatment (n = 1). An additional file includes the list of studies excluded at this stage of the review (see Additional File 1). A further three systematic reviews identified from reference lists were collected, leaving a final number of 31 included in the quality review.

Two authors (CO and AV) then reviewed the included articles against the 11-item AMSTAR (A Measurement Tool to Assess Systematic Reviews) tool for measuring the quality of systematic reviews [18]. The AMSTAR tool was developed following a review of available tools and then further developing and updating them. It was chosen for use in this study because it has been tested and found to be a valid and reliable tool for assessing the quality of systematic reviews [19]. While originally developed and validated for systematic reviews of randomised controlled trials, the tool has recently been validated for systematic reviews of non-randomised studies [20].

**Fig. 1:**
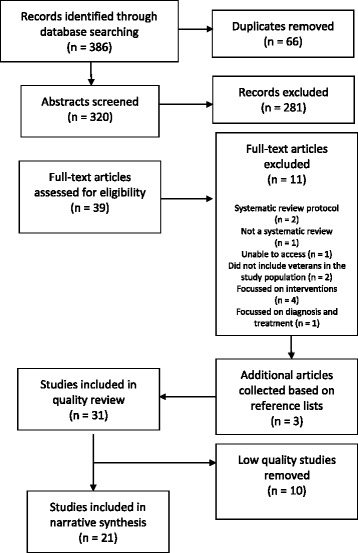
Literature search process and outcome

Following Seo and Kim [21] a score of 0–4 was classified as low quality, 5–8 as moderate quality and 9–11 as high quality. The reviews were of predominantly moderate quality (*n* = 21; 71%), with no high and ten low quality reviews. The ten low quality reviews (scoring ≤4) were removed (these are listed in Additional File 1), leaving a final number of 21 systematic reviews, published between 2003 and 2016, included in the analysis. Figure 1 summarises the literature search process and outcome and Table 1 summarises the literature included in the rapid review.

**Table 1:** Systematic reviews of veterans’ mental, physical and social health

Author (date)	Review methods	Conclusions	AMSTAR rating	Theme(s)
Arriola & Rozelle (2016)	*Eligibility criteria*: English language, peer reviewed publications from 1980 to 2015 reporting quantitative data; *Population*: Adult US OEF/OIF Hispanic veterans; *Outcome*: Traumatic Brain Injury; 34 articles included	• High rates of physical, cognitive, behavioral and emotional symptoms in Hispanic veterans who have suffered TBI • PTSD is strongly associated with TBI	5 (Moderate)	Physical health Mental health Interconnection Factors affecting
Beste & Ioannou (2015)	*Eligibility criteria*: Published between 1992 and April 2014. Excluded: unrelated to the epidemiology of HCV prevalence and treatment, review articles, non–peer-reviewed work, practice guidelines and studies based on case reports/case series. Studies focussing on patients co-infected with HIV and HCV were excluded unless they included an HCV monoinfected arm; *Population*: US veterans aged >18; *Outcome*: Prevalence & treatment of Hepatitis C Virus; 28 articles included	• Approximately 175,000 patients with diagnosed HCV infection are currently in VA care, plus an estimated 45,000 additional patients with as yet undiagnosed infection	6 (Moderate)	Physical health
Blore et al. (2015)	*Eligibility criteria*: Inclusion: (1) The population consisted of military personnel deployed to the Gulf War, Afghanistan or the Iraq War (Army, Navy, Air Force, Marines, Coast Guard, medics, and Reservists/National Guard), (2) published in English, (3) outcome of interest was depression, but studies containing any one of the psychological disorders of depression, anxiety disorders including PTSD, or substance or alcohol use disorders were included, (4) inclusion of an appropriate military comparison group that differed in its level of deployment exposure to the corresponding conflict, (5) enough information to generate an odds ratio (OR) by deployment. Exclusion: (1) The conflict deployed sample was of non-military personnel, (2) published in a language other than English, (3) sample based on clinical or injured or treatment/help-seeking population/s, (4) no appropriate military comparison group included; *Population*: Gulf War, Afghanistan / Iraq War veterans; *Outcome*: Depression; 14 articles included	• Gulf War veterans had over twice the odds of experiencing depression [OR 2.28, 95% CI 1.88–2.76] and dysthymia or chronic dysphoria (OR 2.39, 95% CI 2.0–2.86) compared to non-deployed military personnel	8 (Moderate)	Mental health
Byrne, Montgomery & Dichter (2013)	*Eligibility criteria*: Inclusion: (1) focussed exclusively on female veterans, (2) included specific information about female veterans, and/or (3) involved a comparison of male and female veterans, (4) experimental, observational, descriptive and qualitative studies published in English; Excluded: (1) studies conducted outside of the United States or about veterans who served in the military of a foreign country; *Population*: Female US veterans; *Outcome*: Homelessness; 26 articles included	• Female veterans were at an increased risk of homelessness relative to their male veteran and female non-veteran counterparts • Homeless female veterans were characteristically different from their male counterparts (younger, higher levels of unemployment, lower rates of drug or alcohol dependence or abuse but higher rates of mental health problems) • Risk factors for homeless included unemployment, disability/TBI/poor health, PTSD and other mental health problems, sexual assault/harassment and substance use/abuse	5 (Moderate)	Social health Mental health Physical health Interconnection Factors affecting
Goldzweig et al. (2006)	*Eligibility criteria*: Articles had to describe original research on veterans’ health that either pertained specifically to women veterans, or included explicit information about women veterans or compared men and women veterans; *Population*: Women veterans; *Outcome*: Health; 182 articles included	• Most research on women veterans’ health is descriptive in nature and has concerned PTSD, sexual harassment and assault, the utilisation and organisation of care, and various psychiatric conditions	5 (Moderate)	Mental health Physical health Social health Interconnection Factors affecting
Hoggatt et al. (2015)	*Eligibility criteria*: Inclusion: English-language, peer-reviewed publications reporting on non-tobacco alcohol or drug misuse, abuse, or dependence in US women veterans; published since 1980. Exclusion: studies of women in substance use disorder treatment; clinical trials, studies with nonrepresentative samples (e.g., disease-specific populations), case studies, abstracts, reviews, and commentaries; studies that involved only deceased subjects or examined other addictive disorders; results for women in active duty military service (but included results for members of the reserve forces (National Guard and reserve members); *Population*: US women veterans and National Guard/reserve members; *Outcome*: Alcohol and Drug Misuse, Abuse, and Dependence; 56 articles included	• Rates ranged from 4% to 37% for alcohol misuse and from 7% to 25% for binge drinking • Studies comparing women veterans and civilians reported no clear differences • Substance misuse rates were generally lower among women veterans than men veterans • Substance misuse was associated with higher rates of trauma, psychiatric and medical conditions, and increased mortality and suicide rates	5 (Moderate)	Mental health Physical health Social health Interconnection
Lan et al. (2016)	*Eligibility criteria*: Inclusion: (1) sampled US veterans or reported on US veterans as a separate analytic group, (2) assessed AUDs or DUDs using established DSM or WHO International Classification of Diseases (ICD) diagnostic criteria, (3) reported on the prevalence, distribution, or correlate(s) of SUD(s) in a US veteran sample, (4) published in English in the scientific, peer-reviewed literature. Exclusion: (1) studies that only used screening (rather than diagnostic) tools to identify problematic substance use behaviors, (2) studies that sampled veterans entirely from substance abuse treatment programs and studies that only reported on substance use patterns or frequency (rather than disorders), and did not assess SUDs using established measures, (3) studies published prior to 1987, (4) case reports, case series, editorials, commentaries and previously published narrative reviews. Only included RCTs if pretreatment baseline data on the distribution or correlates of SUDs were reported; *Population*: US veterans; *Outcome*: Substance Use Disorders; 72 articles included	• Relatively high rates of Alcohol Use Disorders (AUD: 32% using diagnostic criteria; 10% using administrative criteria) and Drug Use Disorders (DUD: 20% using diagnostic criteria; 5% using administrative criteria) among US veterans • The observed prevalence of AUD and DUD diagnoses in the sample of veterans were higher than civilians • High rates of PTSD comorbidity with Substance Use Disorders, particularly among veterans with other mental health problems	5 (Moderate)	Mental health Social health Interconnection
MacManus et al. (2015)	*Eligibility criteria*: Inclusion: quantitative studies that 1) operationalised violence and/or aggression as actual physical harm caused by one person against another, a range of aggressive behaviors (property and physical aggression and threat of violence), offending behavior classified as violent, or incarceration for the latter and 2) explored such behaviors among serving or formerly serving military personnel who had been in Iraq or Afghanistan post-2001. Excluded: book chapters, dissertations, case studies, papers published before 2001, qualitative or nonempirical studies, intervention studies, studies reporting a sample size less than 100, articles not in English and studies concerning conflicts other than Iraq and Afghanistan post-2001; *Population*: Serving or formerly serving military personnel who had been in Iraq or Afghanistan post-2001; *Outcome*: Aggressive & Violent Behaviour; 17 articles included	• Reported pooled estimates of 10% (95% CI: 1, 20) for physical assault and 29% (95% CI: 25, 36) for all types of physical aggression in the last month • The majority of studies suggested a small-to-moderate association between combat exposure and postdeployment physical aggression and violence (pooled estimate of the weighted odds ratio = 3.24; 95% CI: 2.75, 3.82) • Violence increased with intensity and frequency of exposure to combat traumas • PTSD plays a mediating role between combat and postdeployment violence and alcohol is a factor, especially if comorbid with PTSD	6 (Moderate)	Social health Mental health Interconnection Factors affecting
Magruder & Yeager (2009)	*Eligibility criteria*: Deployed and non-deployed US Service Personnel, unique studies published in English, with enough information to estimate an odds ratio. Diagnosis of PTSD must be established using a valid and reproducible method, not based on clinical or treatment-seeking populations; *Population*: Active duty military personnel and veterans; *Outcome*: PTSD; 18 articles included	• Odds ratios indicate approximately a 1.5- to 3.5-fold increase in PTSD risk with deployment, regardless of war era • The odds of PTSD for deployed versus non-deployed veterans were lowest among OIF/OEF veterans and highest for Vietnam veterans, with Persian Gulf veterans intermediate	6 (Moderate)	Mental health Factors affecting
McLaughlin, Nielsen & Waller (2008)	*Eligibility criteria*: Peer reviewed articles in English, excluded RCTs, included all other study designs provided that an appropriate control or comparison group (i.e., the general population) was included and reported all-cause, cancer, or external cause mortality; *Population*: Military personal (currently serving & veterans); *Outcome*: Mortality; 12 articles included in the meta-analysis	• The overall healthy soldier effect estimated ranges from 10% to 25%, depending on the cause of death studied and the period of follow-up	7 (Moderate)	Physical health
O’Neil et al. (2014)	*Eligibility criteria*: Studies in English reporting outcomes in Veterans or military personnel with a history of mTBI (using a case definition consistent with VA/DoD Clinical Practice Guideline for Management of Concussion/Mild Traumatic Brain Injury); report health or cost outcomes for members of the US armed forces or Veterans; Eligible study designs: systematic reviews; meta-analyses; randomized controlled trials; and cohort, case-control, cross-sectional, or case series studies, with a minimum of 30 mTBI cases.; *Population*: Veterans or military personnel with a history of mTBI; *Outcome*: Factors associated with mTBI; 31 articles included	• Cognitive, physical, and mental health symptoms were commonly reported by Veterans/military members with a history of mTBI • Findings are consistent with civilian studies	5 (Moderate)	Physical health Mental health Social health Interconnection
Pompili et al. (2013)	*Eligibility criteria*: English language, peer reviewed articles, from 1980; excluded articles without abstracts, abstracts that did not explicitly mention suicidal behavior and war related PTSD; *Population*: Veterans; *Outcome*: PTSD and suicide risk; 18 articles included	• Having a history of PTSD is associated with higher rates of morbidity and mortality and increased the risk for suicidal behavior • The association between PTSD and suicidal behavior was confirmed by the presence of other risk factors and high rates of comorbidity	5 (Moderate)	Mental health Physical health Interconnection
Stevelink et al. (2015)	*Eligibility criteria*: Comprised data on (ex-)military personnel with a physical, visual or hearing impairment; administered at least one validated outcome measure of mental health or participants self-reported to have a mental health problem, or hospital records/military databases indicated the presence of a mental health problem; were reported in English; *Population*: (Ex-)military personnel with a physical impairment; *Outcome*: Mental health disorders; 25 articles included	• Mental health disorders including PTSD (range 2–59%), anxiety (range 16.1–35.5%), depression (range 9.7–46.4%) and psychological distress (range 13.4–36%) are frequently found in veterans with a physical impairment • Alcohol misuse was least common (range 2.2–26.2%)	6 (Moderate)	Mental health Physical health Social health Interconnection
Stimpson et al. (2003)	*Eligibility criteria*: Contained data on veterans who had been deployed to the Gulf War on military, medical or peacekeeping grounds; any study design was eligible for inclusion provided that an appropriate control or comparison group was included to compare the prevalence of psychiatric disorder; *Population*: Veterans of the Persian Gulf War of 1991; *Outcome*: Psychiatric disorder; 20 articles included	• Prevalence of PTSD and common mental disorder were higher in the Gulf War veterans than active service personnel not deployed to the Gulf	8 (Moderate)	Mental health Social health Factors affecting
Theodoroff et al. (2015)	*Eligibility criteria*: Studies involving US veterans and military service members who served in OEF/ OIF/ OND; original data, minimum sample size of 30, or systematic reviews. Excluded: Narratives, letters, editorials, commentaries; not address the key questions or differentiate between who did versus did not serve in OEF/ OIF/ OND or between veteran/military and civilian populations. *Population*: US Service Members and Veterans Deployed to the Iraq and Afghanistan Wars; *Outcome*: Hearing Impairment and Tinnitus; 14 articles included	• Auditory complaints are highly prevalent; in some injured populations, greater than 50% • Among more than 90,000 veterans seen in the VHA system of care 16.4%– 26.6% of men and 7.3%– 13.4% of women were diagnosed with hearing problems • Risk/protective factors appear to include age, gender, military branch and component, blast exposure, PTSD diagnoses, and characteristics of initial injuries	5 (Moderate)	Physical health Mental health Interconnection Factors affecting
Thomas et al. (2006a)	*Eligibility criteria*: Published between January 1990 and May 2004; included if they reported the prevalence of any symptom or condition that included the word “pain” in Gulf War veterans and in a comparison group of non-Gulf veterans. Excluded: measured simulated exposures/ non-health related outcomes; subjects were inhabitants of the Persian Gulf rather than deployed military personnel; examined pain within groups of Gulf veterans that had experienced differential exposures whilst in the Gulf; *Population*: Gulf War veterans; *Outcome*: Pain; 20 articles included	• A greater proportion of Gulf veterans reported symptoms at each of five main sites of pain (muscle, joint, chest/ heart, back and abdominal pain) when compared to a non-Gulf military group • Gulf deployment was most strongly associated with abdominal pain (Gulf veterans more than three times more likely to report such pain than a comparison group (OR 3.23; 95%CI 2.31–4.51))	8 (Moderate)	Physical health Factors affecting
Thomas et al. (2006b)	*Eligibility criteria*: Published between January 1990 and May 2004; included if they compared the prevalence of chronic fatigue syndrome, multiple chemical sensitivity, CDC-defined chronic multi-symptom illness, fibromyalgia, or symptoms of either fatigue or numbness and tingling in Gulf War veterans and non-Gulf veterans; *Population*: Gulf War veterans; *Outcome*: Multi-symptom conditions; 23 articles included	• Gulf deployment most strongly associated with chronic fatigue syndrome (OR 3.8, 95% CI 2.2–6.7) • Gulf War veterans were also approximately three and a half times more likely than non-Gulf veterans to report multiple chemical sensitivity or chronic multi-symptom illness as defined by CDC	8 (Moderate)	Physical health Factors affecting
Tsai & Rosenheck (2015)	*Eligibility criteria*: Published in English from 1900 to July 2014; sampled US veterans; assessed homelessness in the US; included homelessness as an outcome or dependent variable; examined variables in relation to homelessness as a main study aim with the intent to identify risk factors / characteristics associated with homelessness. Excluded: reported only the effects of a specific intervention / reported only qualitative data; case reports, published commentaries, and letters to the editor that did not report any quantitative data; *Population*: US veterans; *Outcome*: Homelessness; 31 articles included	• The strongest and most consistent risk factors for homelessness in US veterans were substance use disorders and mental illness, followed by low income and other income-related factors • Veterans were at greater risk for homelessness than other adults	7 (Moderate)	Mental health Social health Interconnection Factors affecting
Wall (2012)	*Eligibility criteria*: Published in English between 2001 and 2011. Excluded: individual case studies; studies presented as meeting abstracts; studies involving veteran populations from conflicts other than OEF/OIF/OND; studies not measuring both PTSD and TBI; those involving civilian populations or non–U.S. military populations; *Population*: US OEF/OIF military and veteran populations; *Outcome*: PTSD and TBI; 20 articles included	• There is some evidence that comorbid PTSD and TBI result in greater reports of postconcussive symptomology than either condition alone • Rates of PTSD vary between 11% and 79%, rates of TBI vary between 4.9% and 41%, and comorbid rates of PTSD and TBI vary between 0.02% and 26%	6 (Moderate)	Mental health Physical health Interconnection
Wright et al. (2013)	*Eligibility criteria*: Published in English between January 1990 and October 2011; any study design; participants had been deployed to the Gulf War (1990–1991), the Afghanistan conflict (since 2001), or the Iraq War (since 2003); any deployed persons (army, navy, air force, marines, medics); reservists or regular military personnel; the outcome of focus was PTSD; diagnosis was defined through doctor diagnosis, self-report, health data linkage, or validated assessment tools. At least one of the following variables was included: unit cohesion or perceptions of unit cohesion, social support or perceptions of social support, family support or perceptions of family support, personality trait of neuroticism, or psychiatric history. Excluded: studies that looked at psychological outcomes which were not PTSD or PTSD as a psychological comorbidity; participant populations of contractors or nonmilitary personnel; *Population*: Veterans of the Gulf War, Iraq War, and Afghanistan Deployments; *Outcome*: Support mechanisms and vulnerabilities in relation to PTSD; 17 articles included	• Low unit cohesion was associated with PTSD, standardised mean difference of −1.62, 95% confidence interval (CI) [−2.80, −0.45] • Low social support was associated with PTSD, standardised mean difference of −12.40, 95% CI [−3.42, −1.38] • Significant relationship between low-family support and PTSD (insufficient data precluded a meta-analysis) • Posttrauma factors of low support were associated with higher reporting of PTSD	5 (Moderate)	Mental health Social health Interconnection Factors affecting
Xue et al. (2015)	*Eligibility criteria*: Investigated risk factors for PTSD in military populations after deployment to combat areas; reported the odds ratios or relative risks and corresponding 95% confidence intervals for risk factors in the development of PTSD; included the selected postdeployment PTSD risk factors; included a sample of military personnel, veterans, or both. Excluded: measured only the acute trauma response rather than PTSD; used a categorical measure of PTSD; the study population consisted entirely of individuals already suffering from PTSD or from a specific comorbid psychiatric disorder or having committed a violent offense; did not specifically assess DSM-defined PTSD symptoms; contained insufficient data to calculate univariate effect sizes, and such data could not be obtained from the study author; review or qualitative studies not presenting new data or only presented qualitative analyses; primary aim to investigate the efficacy of treatment; the study used a single-case design; *Population*: Military personnel and veterans; *Outcome*: Risk factors for combat-related PTSD; 32 articles included	• The prevalence of combat-related PTSD ranged from 1.09%to 34.84% • Risk factors stemming from before the trauma include female gender, ethnic minority status, low education, non-officer ranks, army service, combat specialisation, high numbers of deployments, longer cumulative length of deployments, more adverse life events, prior trauma exposure, and prior psychological problems • Risk factors relating to aspects of the trauma period include increased combat exposure, discharging a weapon, witnessing someone being wounded or killed, severe trauma, and deployment-related stressors • Lack of post-deployment support during the post-trauma period also increased risk of PTSD	7 (Moderate)	Mental health Physical health Interconnection Factors affecting

### Description of the included literature

Five reviews focussed on veterans in general. Other reviews focussed on veterans of particular wars, including the Gulf War (*n* = 5) and the recent Iraq and Afghanistan conflicts (Operation Enduring Freedom (OEF)/Operation Iraqi Freedom (OIF); *n* = 6), and veterans from particular countries (US: *n* = 8). Three reviews focussed on female veterans.

As per Khangura et al.’s [16] rapid review process, a narrative synthesis of the reviews was developed. This involved categorising the reviews into themes. The themes reflect the rapid review questions devised in consultation with the DVA (‘What are the mental, physical and social wellbeing needs of veterans?’; ‘What are the factors associated with the mental, physical and social wellbeing needs of veterans?’), in addition to the theme ‘Interconnection between wellbeing areas’. The themes are described in Table 2.

**Table 2:** List of themes

Theme	Description
Mental wellbeing	Relating to diseases and disorders of the mind
Physical wellbeing	Relating to physical functioning (e.g., disability), diseases and disorders of the body, and pain
Social wellbeing	Relating to social activities/networks, relationships, substance use/abuse (drugs, alcohol, and smoking), housing and employment/financial wellbeing. [Note: We have defined substance use/abuse as a social health issue because it exists within a social context, affects the social environment surrounding the person, and arises from the social and cultural context]
Interconnection between wellbeing areas	Articles reporting an interconnection or association between physical, mental, and/or social wellbeing (i.e., between any two or all three)
Factors affecting wellbeing	Articles reporting on factors affecting the physical, mental and/or social wellbeing of veterans

All of the included systematic reviews were independently reviewed by two authors (CO and SL) and assigned to the identified themes, with disagreements discussed and resolved. The number of reviews categorised into each of the identified themes is summarised in Table 3.

**Table 3:** Number of systematic reviews categorised into each of the identified themes

Theme	Number of Articles (% of the total 21 included in the review)
Mental health	17 (81%)
Physical health	14 (67%)
Social health	10 (48%)
Interconnection between health areas	14 (67%)
Factors affecting	12 (57%)

The literature is described below within each identified theme.

### Veterans’ mental wellbeing

The majority of the articles discussed the mental wellbeing of veterans (*n* = 17; 81%). Mental health research relating to veterans has a strong focus on PTSD. Wall [22] found rates of PTSD in veteran populations varied between 11% and 79%. Focussing on combat-related PTSD, Xue et al. [23] found the prevalence ranged from 1.09% to 34.84%. Variations in rates are likely due to differences across studies in their design, sampling and measurement [22].

The systematic reviews provided limited information about the prevalence of mental health problems in veterans outside of the experience of PTSD. None of the systematic reviews reported on the prevalence estimates of depression, anxiety, psychotic or other mental health disorders in the general population of veterans, nor compared rates of mental health problems in veterans to rates in civilian populations. They did, however, report on mental wellbeing in relation to deployment status [24] and in association with, and as a risk factor for, physical and social wellbeing, discussed later.

### Veterans’ physical wellbeing

Fourteen articles discussed the physical wellbeing of veterans (*n* = 14; 67%). According to one review veterans do display a healthy soldier effect, with veterans’ mortality rates being 10% to 25% lower than the general population [25]. The review evaluated the effect of military service on mortality by comparing the mortality of veterans to that of the general (civilian) population using the Standard Mortality Ratio (SMR; used to compare the mortality of the group in question with that of the general population).

Despite the healthy soldier effect, veterans are still at risk of serious injury and illness relating to their military life. However, as with the reviews relating to veterans’ mental wellbeing, reviews discussing physical wellbeing provided limited information on prevalence rates in the general population of veterans or comparisons to civilian populations. Rates of particular physical health problems explored in the reviews included TBI, hearing impairment/tinnitus and Hepatitis C Virus.

Three systematic reviews explored TBI, only one of which reported prevalence rates between 4.9% and 41% of US OEF/OIF veterans [22]. The other two reviews did not examine TBI prevalence but did indicate that TBI is associated with a wide range of health problems such as headaches, pain, vestibular outcomes, vision-related outcomes, hearing related outcomes, neurological outcomes and outcomes related to appetite and nausea [26, 27].

A review of hearing impairment and tinnitus in veterans of the OEF/OIF conflicts [28] found 16.4%–26.6% of men and 7.3%–13.4% of women were diagnosed with hearing problems. Rates of auditory complaints in some injured OEF/OIF veteran populations were greater than 50%. No information on rates in veterans of other wars was presented.

Finally, one review reported on the prevalence of chronic Hepatitis C Virus in US Department of Veterans Affairs’ (VA) health-care users [29]. Based on a descriptive review of the literature, the authors reported approximately 175,000 (3%) VA patients with diagnosed HCV infection and an estimated 45,000 (0.8%) undiagnosed patients. The review did not include a comparison with civilian populations or veterans from other countries. Further information on veterans’ physical wellbeing will be discussed in relation to associations with, and risk factors for, mental and social wellbeing.

### Veterans’ social wellbeing

Ten articles discussed the social wellbeing of veterans (*n* = 10; 48%). Overall, the literature describes veterans as being at greater risk of substance use/misuse and homelessness than civilians. For example, Lan et al. [30] reported relatively high rates of alcohol use disorders (32% using diagnostic criteria; 10% using administrative criteria) and drug use disorders (20% using diagnostic criteria; 5% using administrative criteria) in US veterans. The observed prevalence was higher than civilians but showed a gradual decline over time. Hoggatt et al. [31] reported rates ranging from 4% to 37% for alcohol misuse and from 7% to 25% for binge drinking in female veterans. There were no clear differences identified in rates between female veterans and civilians.

Three reviews reported on the issue of homelessness in veteran populations [32–34]. No information on rates of homelessness in veterans in general were reported, but two studies reported prevalence rates for homelessness in female veterans, with rates ranging from 1% to 2% of all female veterans [32, 33]. In addition, female veterans were 2.1–2.5 times as likely as women in the general population to be homeless [32]. Tsai and Rosenheck [34] reported that veterans in general were at greater risk for homelessness than civilian populations, but did not provide prevalence rates for this statement.

In addition to substance abuse/misuse and homelessness, one study reviewed aggressive and violent behaviour in veterans. Pooled estimates of 10% for physical assault and 29% for physical aggression in the last month were reported [35]. No information on rates in civilian populations was included. Further information on veterans’ social wellbeing will be discussed in relation to associations with, and risk factors for, mental and physical wellbeing.

### Interconnection between wellbeing areas

A key issue identified in the systematic reviews is the interconnection between the three wellbeing areas discussed above. The interconnection between veterans’ physical wellbeing, mental wellbeing and social wellbeing was discussed in 14 (67%) reviews. Five reviews reported an interconnection between physical and mental wellbeing, four between mental and social wellbeing, and five between all three areas.

The most commonly reported interconnection was related to PTSD. A diagnosis of PTSD was reported to increase veterans’ risk of physical health problems [33, 36]; substance use/ misuse [30, 31]; suicide [36]; homelessness [32, 34]; and aggression/ violence [35]. Associations were also reported between PTSD and TBI [26, 27], and between PTSD and hearing impairment and tinnitus [28]. Lack of social support, including support from peers, family and spouse/ partner, was also discussed as a risk factor for PTSD [37].

The interconnection between other mental health problems, such as anxiety and depression, and physical wellbeing was also reported [26, 27]. For example, veterans with physical impairment were reported to have high rates of PTSD (2–59%), anxiety (16.1–35.5%), depression (9.7–46.4%) and psychological distress (13.4–36%) [38]. Mental health problems were also associated with homelessness [32, 34] and substance use/ abuse [33]. In addition, homelessness was associated with physical disability and poor health status [32] and substance use/ misuse [34]. Hoggatt et al. [31] found substance misuse to be associated with higher rates of trauma, psychiatric and medical conditions, and increased mortality and suicide rates. Low social support was also reported as a risk factor for homelessness [34].

More complex interconnections between the various domains were also discussed. For example, Lan et al. [30] found PTSD comorbidity with substance use disorders to be particularly high among veterans with other mental health problems. Wall [22, p.278] reported that there is “some evidence that comorbid PTSD and TBI result in greater reports of postconcussive symptomology than either condition alone”. Finally, Godzweig et al. [33] reported an association between trauma (in the form of sexual harassment, abuse and assault) and poorer health status and higher rates of medical and psychiatric conditions in female veterans.

### Factors affecting the physical, mental, and/or social wellbeing of veterans

In addition to the reported interconnection between the various attributes of veterans’ wellbeing, eleven reviews (57%) reported on the factors affecting the physical, mental and/ or social health of veterans. As the previous section suggests, problems in one or more of these areas are themselves risk factors for problems in other areas. Other key factors affecting the health and wellbeing of veterans are factors related to veterans’ military service and demographic factors.

#### Factors related to veterans’ military service

The most commonly identified factor related to military service was the effect of deployment on veterans’ wellbeing. Aspects of deployment included whether or not the veteran was deployed, the number of deployments and the location of deployment. Deployment, in particular multiple deployments, was associated with an increased risk for PTSD and other mental health problems [23, 24, 33, 39, 40], hearing impairment/ tinnitus [28], pain [41], multi-symptom conditions [42], and aggressive and violent behaviours [35]. However, McLaughlin et al. [25] found no differences in the healthy soldier effect in deployed compared with non-deployed veterans.

The location of deployment was another factor reported to affect veterans’ wellbeing. Deployment to the Gulf War was explored in the three systematic reviews, all three reporting Gulf War veterans to be at greater risk of physical and mental health problems than veterans of other wars. Blore et al. [24] found Gulf War veterans to be more than twice as likely as non-Gulf War veterans to experience depression, dysthymia and chronic dysphoria. In a review comparing pain in Gulf War compared to non-Gulf War veterans [41], a higher proportion of Gulf veterans reported pain at each of the five main sites of pain identified in the review (muscle, joint, chest/ heart, back and abdominal pain), with abdominal pain being most strongly associated with Gulf War deployment. Gulf War deployment was also associated with greater reporting of multi-symptom conditions (chronic fatigue syndrome, multiple chemical sensitivity, CDC-defined chronic multi-symptom illness, fibromyalgia, or symptoms of either fatigue or numbness and tingling) compared to non-Gulf War deployment [42].

The experience of trauma during deployment also affected veterans’ health. Exposure to combat reportedly increased veterans’ risk of PTSD [23, 36], and post deployment physical aggression and violence [35]. Military sexual trauma (sexual assault or harassment) also increased female veterans' risk of PTSD and other psychiatric conditions [33], homelessness [32] and increased numbers of medical conditions [33].

Another important service-based factor discussed in the literature relates to support. Service-based support in the form of unit cohesion and post-deployment support were discussed in two reviews [23, 37]. Low unit cohesion and lack of post deployment support were reported as risk factors for PTSD in these reviews.

Finally, military rank and branch were also discussed. Xue et al. [23] found an association between PTSD and non-officer ranks. Theodoroff et al. [28] reported an association between hearing impairment/ tinnitus and military branch (e.g., army, navy, air force) and the unit within the branch.

#### Demographic factors

In addition to service-based factors, demographic factors were also reported to affect the health and wellbeing of veterans. Gender was the most commonly discussed factor, reported in five reviews. For example, the reviews identified that female veterans experienced more mental health problems than men, including panic disorder, depression and other anxiety disorders [33], and PTSD [23, 33].

In addition, Byrne et al. [32] reported that there is some evidence that female veterans have a higher risk of homelessness than male veterans. Female veterans who were homeless were also characteristically different from males (younger, with higher levels of unemployment, lower rates of drug or alcohol dependence or abuse, and higher rates of mental health problems). By contrast, female veterans were reported to be at lower risk than male veterans for substance use/ misuse [31] and hearing problems [28].

The impact of age on veterans’ health was reported in four reviews. Older age was associated with homelessness in female veterans [32], with Tsai and Rosenheck [34] also reporting that veterans who were homeless were generally older than other adults who were homeless. Younger age was associated with an increased risk of being diagnosed with a psychiatric disorder in female veterans [33]. In addition age was associated with hearing loss and tinnitus ([28]; although the review did not report on the direction of the association).

Factors reported less frequently included unemployment/ low income, education and ethnicity. Ethnicity was discussed in two reviews; Arriola & Rozelle [26] found high rates of physical, cognitive, behavioral and emotional symptoms in Hispanic veterans with TBI, and Xue et al. [23] reported ethnic minority status to be associated with PTSD. Being unemployed or on a low income increased the risk of homelessness [32, 34]. Finally, Xue et al. [23] found low education to be associated with PTSD.

## Discussion

In this rapid review of the literature we have explored the wellbeing needs of veterans within the context of their broader mental, physical and social determinants of health. We examined the findings of 21 moderate quality systematic reviews exploring a wide range of issues. High levels of effective general functioning are reported in studies of veteran populations [63,64]. However, as this rapid review demonstrates, a large number of veterans still experience mental, physical and social health problems related to life after the military, with the associated health care costs that come with this. The current review sought to better understand these needs to provide policy makers, service providers and healthcare professionals with an understanding of veterans’ wellbeing encompassing all three attributes of wellbeing [15]. Understanding and recognising the interconnectedness across these attributes may facilitate the early identification and improved management of veterans’ health.

The mental health literature demonstrates high rates of PTSD in veteran populations. The literature furthermore highlights the worrying interconnection between PTSD and the increased risks for veterans of physical health problems, substance use/ misuse, suicide, homelessness and aggression/ violence. However, the literature fails to adequately report on prevalence rates of mental health problems other than PTSD, and in particular to compare these rates to the general (civilian) population. Veterans clearly experience a range of mental health problems in addition to PTSD, particularly anxiety and depression, and comorbidy of mental health problems. However, as Blore et al. [61, p.1577] noted in their review of depression in Gulf War veterans, “[t]he reality is that depression is often ignored in studies of veterans’ mental health, which tend to focus on PTSD”. The same can be said for anxiety and other mental health problems, and more systematic reviews focussing on these issues in the general population of veterans are needed, along with comparisons of rates between veteran and civilian populations.

When considering the physical wellbeing of veterans, McLaughlin et al.’s [25] review supports the notion of a healthy soldier effect (although a review of more recent research is needed, given reports of a reduced healthy soldier effect in veterans of recent conflicts [9]). However, the literature also demonstrates a range of physical health problems directly related to veterans’ previous military role, particularly traumatic brain injury, hearing impairment, tinnitus, pain and multi-symptom conditions. Veterans’ physical health also interconnects with their mental and social wellbeing, such as the increased prevalence of mental health disorders in veterans with a physical impairment [38] and the association of homelessness with physical disability and poor health status [32] and substance use [34]. While other studies have reported that veterans experience low levels of general health and health related quality of life [43–45] and increased risk of physical disability and chronic illness [46–49], no systematic reviews focussing on these issues in veterans were identified.

The literature describing veterans’ social health furthermore articulates the potential difficulties of transitioning to civilian life post-military service [13, 50]. Problems reported in the literature included substance use/ misuse, homelessness, inadequate social support, issues relating to financial wellbeing and aggressive behaviours. These problems point to difficulties in adjusting to the less stable social variables that veterans might encounter when leaving the structured military environment. Of particular importance is the interconnection of veterans’ social health, principally homelessness and substance use/ misuse, with veterans’ mental and physical wellbeing. The literature on homelessness in veteran populations demonstrates the compounding of risk when several issues (in particular substance misuse disorders, mental illness and low income) are present [34].

When considering the literature describing the various factors associated with veterans’ mental, physical and social wellbeing, demographic factors in particular could help identify those veterans who might be at greater risk (though the question of why is still open for more research). There has been an increase in studies focussing on the issues facing female veterans, reflecting increasing numbers of women in the military and engaged in conflicts [51]. Issues of particular concern for female veterans include reported higher rates of PTSD and military sexual trauma than male veterans, and greater effects of these issues on women’s mental, physical and social wellbeing. The studies of service type add a further layer of understanding to the scale and complexity of risk by examining the influence of the actual soldier role and experience.

When serving in the military, individuals are provided with structured, comprehensive health care; care that is often no longer available after transitioning to civilian life, unless the veteran has been assessed as having ongoing healthcare needs related to their military service. Research with Vietnam veterans, for example, has shown that many do not have their healthcare needs recognised until several years after their return to civilian life, for a range of reasons including delays in help-seeking and delayed onset (for example, PTSD) [52]. The results of this rapid review, and in particular the identification of the interconnection between veterans’ physical, mental, and social wellbeing, suggests a need for the provision of veterans’ health care that is streamlined and integrated across these areas. This is likely important for prevention and early identification of veterans at risk of developing problems in any or all of these three areas.

Veterans’ reported reluctance to seek help, particularly for mental health problems [53, 54], furthermore highlights the importance of policy makers, service providers and health care professionals having a structured process by which they can comprehensively assess and support the health care needs of this unique population. For example, in a recent paper by Reed et al. [55], the authors introduced the Australian Defence Force Post-discharge GP Health Assessment. This structured assessment aims to promote early detection and intervention across the mental, physical and social health domains in veteran populations, with the aim of helping former members of the defence force to access primary health care after transitioning to civilian life. Further research on the impact of this type of assessment is needed [55].

## Limitations

Being a rapid review of the literature, this paper is by definition limited by its short timeframe [16]. However, it has included a large number of systematic reviews covering a broad range of veterans’ physical, mental and social care needs. The systematic reviews were of a moderate quality according to the AMSTAR ratings, with no high quality systematic reviews identified. This could relate to the quality of the studies themselves, or to the application of the AMSTAR assessment tool, designed for systematic reviews of randomised controlled trails, to reviews of both randomised and non-randomised studies. While the tool has been validated for application to these studies, showing good psychometric properties [20], the items on the tool most responsible for lower scores (no a priori design provided, failure to include potential sources of support for both the systematic review and for each of the included studies, and failure to include a list of excluded studies) are perhaps less commonly included in reviews of non-randomised studies.

## Conclusions

In this rapid review we have described the broad range of veterans’ mental, physical and social health care needs, in addition to the factors that affect the health and wellbeing of veterans. A key finding of the review is the interconnection between veterans’ physical, mental and social wellbeing. The findings therefore support the value of integrating veterans’ health care across the domains of physical, mental and social health in order to ensure that members of the military are taken care of both inside and outside of the uniform.

## Additional file

Additional file 1: List of excluded studies. (DOCX 98 kb)

## Abbreviations

AMSTAR: A Measurement Tool to Assess Systematic Reviews
CDC: Centers for Disease Control
DVA: Department of Veterans' Affairs (Australia)
HCV: Hepatitis C Virus
OEF: Operation Enduring Freedom
OIF: Operation Iraqi Freedom
PTSD: Post Traumatic Stress Disorder
SMR: Standard Mortality Ratio
TBI: Traumatic Brain Injury
UK: United Kingdom
US: Unites States
VA: Veterans Affairs (United States)
WHO: World Health Organization
