# Supplementary material for: The health and wellbeing needs of veterans: a rapid review
Source: BMC Psychiatry. 2017 Dec 29;17:414. doi: 10.1186/s12888-017-1547-0 (PMC5747125; doi:10.1186/s12888-017-1547-0)
Supplement: Additional file 1: — List of excluded studies. (DOCX 98 kb) [file 12888_2017_1547_MOESM1_ESM.docx]

**Additional File 1**

***List of excluded studies***

Caddick N, Smith B. The impact of sport and physical activity on the well-being of combat veterans: a systematic review. Psychol Sport Exerc. 2014;159–18.

Cooper DB, Bunner AE, Kennedy JE, Balldin V, Tate DF, Eapen BC, et al. Treatment of persistent post-concussive symptoms after mild traumatic brain injury: a systematic review of cognitive rehabilitation and behavioral health interventions in military service members and veterans. Brain Imaging Behav. 2015;9:403–20.

Goetter EM, Bui E, Ojserkis RA, Zakarian RJ, Brendel RW, Simon NM. A systematic review of dropout from psychotherapy for posttraumatic stress disorder among Iraq and Afghanistan combat veterans. J Trauma Stress. 2015;28:401–9.

Ivanov I, Yehuda R. Optimizing fitness for duty and post-combat clinical services for military personnel and combat veterans with ADHD: a systematic review of the current literature. Eur J Psychotraumatol. 2014;5:23894.

Moon A, Lawson K, Carpiac M, Spaziano E. Elder abuse and neglect among veterans in Greater Los Angeles: prevalence, types, and intervention outcomes. J Gerontol Soc Work. 2006;46:187–204.

Orner RJ. Post-traumatic stress disorders and European war veterans. Br J Clin Psychol. 1992;31:387–403.

Salamati P, Razavi SM, Shokraneh S, Mohazzab Torabi S, Laal M, Hadjati G, et al. Mortality and injuries among Iranians in Iraq-Iran War: a systematic review. Arch Iran Med. 2013;16:542–50.

Taylor J, Parkes T, Haw S, Jepson R. Military veterans with mental health problems: a protocol for a systematic review to identify whether they have an additional risk of contact with criminal justice systems compared with other veterans groups. Syst Rev. 2012;1:53.

Trevillion K, Williamson E, Thandi G, Borschmann R, Oram S, Howard LM. A systematic review of mental disorders and perpetration of domestic violence among military populations. Soc Psychiatry Psychiatr Epidemiol. 2015;50:1329–46.

Van Denkerkhof EG, Carley ME, Hopman WM, Ross-White A, Harrison MB. Prevalence of chronic pain and related risk factors in military veterans: a systematic review. JBI Database of System Rev Implement Rep. 2014;12:152–86.

Van Til L, Fikretoglu D, Pranger T, Patten S, Wang JL, Wong M, et al. Work reintegration for veterans with mental disorders: a systematic literature review to inform research. Phys Ther. 2013;93:1163-74.

***Studies excluded because of low AMSTAR scores***

Bartlett BA, Mitchell KS. Eating disorders in military and veteran men and women: a systematic review. In J Eat Disord. 2015;48:1057-69.

Bean-Mayberry B, Yano EM, Washington DL, Goldzweig C, Batuman F, Huang C, et al. Systematic review of women veterans’ health: update on successes and gaps. Womens Health Issues. 2011.

Bentsen IL, Giraldi AG, Kristensen E, Andersen HS. Systematic review of sexual dysfunction among veterans with post-traumatic stress disorder. Sex Med Rev. 2015;3:78-87.

Blodgett JC, Avoundjian T, Finlay AK, Rosenthal J, Asch SM, Maisel NC, et al. Prevalence of mental health disorders among justice-involved veterans. Epidemiol Rev. 2015;37:163-76.

Chang ET, Boffetta P, Adami HO, Cole P, Mandel JS. A critical review of the epidemiology of Agent Orange/TCDD and prostate cancer. Eur J Epidemiol. 2014;29:667-723.

Middleton K, Craig CD. A systematic literature review of PTSD among female veterans from 1990 to 2010. Soc Work Ment Health. 2012;10:233-52.

Patterson AT, Kaffenberger BH, Keller RA, Elston DM. Skin diseases associated with Agent Organge and other organochlorine exposures. J Am Acad Dermatol. 2016;74:143-70.

Razavi M, Salamati P, Saghafinia M, Abdollahi M. A review on delayed toxic effects of sulphur mustard in Iranian veterans. Daru. 2012;20:51.

Rozanov V, Carli V. Suicide among war veterans. Int J Environ Res Public Health. 2012;9:2504-19.

Runnals JJ, Garovoy N, McCutcheon SJ, Robbins AT, Mann-Wrobel MC, Elliott A, et al. Systematic review of women veterans’ health. Womens Health Issues. 2014;24:485-502.
